# Association between Rheumatoid Arthritis and Meniere’s Disease: A Longitudinal Follow-Up Study Using a National Health Screening Cohort

**DOI:** 10.3390/jcm10235694

**Published:** 2021-12-03

**Authors:** So Young Kim, Dae Myoung Yoo, Ji Hee Kim, Mi Jung Kwon, Joo-Hee Kim, Hyo Geun Choi

**Affiliations:** 1 Department of Otorhinolaryngology-Head & Neck Surgery, CHA Bundang Medical Center, CHA University, Seongnam 13496, Korea; sossi81@hanmail.net; 2 Hallym Data Science Laboratory, Hallym University College of Medicine, Anyang 14068, Korea; ydm1285@naver.com; 3 Department of Neurosurgery, Hallym University College of Medicine, Anyang 14068, Korea; kimjihee.ns@gmail.com; 4 Department of Pathology, Hallym University College of Medicine, Anyang 14068, Korea; mulank@hanmail.net; 5 Division of Pulmonary, Allergy, and Critical Care Medicine, Department of Medicine, Hallym University College of Medicine, Anyang 14068, Korea; luxjhee@gmail.com; 6 Department of Otorhinolaryngology-Head & Neck Surgery, Hallym University College of Medicine, Anyang 14068, Korea

**Keywords:** Meniere’s disease, rheumatoid arthritis, case-control studies, cohort studies

## Abstract

This study aimed to evaluate the impact of pre-existing rheumatoid arthritis (RA) on the occurrence of Meniere’s disease (MD). The 2002–2015 Korean National Health Insurance Service—Health Screening Cohort data were retrospectively analyzed. A total of 3038 participants with RA were matched with 12,152 control participants for demographic factors. The occurrence of MD was evaluated in both the RA and control groups. The hazard ratios (HRs) of RA for participants with MD were calculated using a stratified Cox proportional hazard model. Additionally, subgroup analyses were conducted. The rate of MD was not different between the RA and control groups (1.5% vs. 1.3%, standardized difference = 0.01). The HR was not higher in the RA group than in the MD group (adjusted HR = 1.03, 95% confidence interval = 0.73–1.44, *p* = 0.885). A higher HR of RA for participants with MD was found in the ≥60-year-old subgroup in the crude model but not in the adjusted model. An association between RA and MD was not found in any of the other subgroups. A previous history of RA was not related to an increased risk of MD.

## 1. Introduction

Rheumatoid arthritis (RA) is a systemic inflammatory disease that primarily involves the joints [1]. Although the prevalence of RA varies according to ethnicity, it is estimated to affect approximately 0.5–2% of the general population [2]. The pathophysiology of RA is multifactorial and includes genetic, autoimmune, and environmental factors [3,4]. Inflammation of the synovial space induces proinflammatory cytokines such as tumor necrosis factor and interleukin-6, which proliferate in synovial cells or pannus and derange cartilage and bony structures [3]. According to the progression of RA, an increasing number of joints are affected by inflammatory changes and develop symptoms of pain and stiffness [4]. In addition to multijoint involvement, RA has been acknowledged to cause extra-articular manifestations, such as rheumatoid nodules and pulmonary, cardiac, and ocular manifestations [5]. Furthermore, the increased risk of falls and vertigo in patients with RA has been attributed to multijoint damage and systemic manifestations [6]. As many as 52.2% of patients with RA reported falls, and vertigo was associated with a higher risk of recurrent falls (odds ratio = 3.42, *p* = 0.036) [6].

Meniere’s disease (MD) is a cochleovestibular disorder diagnosed based on the relapsing symptoms of hearing loss, ear fullness, tinnitus, and vertigo [7]. The prevalence of MD was estimated to be approximately 17.0–70.4 cases per 100,000 people [8,9]. The pathophysiology of MD has been explained by several mechanisms, including autoimmunity, inflammation, environmental factors, and genetic factors [10,11,12]. In particular, the contribution of autoimmunity has been suggested, and MD is considered an autoimmune inner ear disease [13,14]. Compared to a control group, patients with MD showed elevated levels of serum circulating immune complexes (absolute level of optical density at 450 nm * 10^3^/mL = 1445 ± 577 vs. 470 ± 149, *p* < 0.001) [14]. In addition, a randomized controlled trial demonstrated significant improvement in Dizziness Handicap Inventory scores (60.4 vs. 41.3) following intratympanic steroid treatment in patients with MD [15]. Because both MD and RA share a common pathophysiology of autoimmunity, the association of autoimmune arthritis, including RA, with MD has been proposed [13]. However, the prevalence of autoimmune arthritis in patients with MD varies from 1.0% to 10.0%, and the study population was not standardized to a control population [13].

We hypothesized that a prior history of RA could impact the subsequent occurrence of MD. However, comorbid conditions could influence the association of RA with MD. In addition, the vertigo symptoms in patients with RA could originate from other vestibular disorders. To test this hypothesis, patients with RA were compared with a control group for the new onset of MD. To minimize the potential confounding effects, data on comorbidities, including other vestibular disorders, were collected, and comorbidities were adjusted as covariables.

## 2. Materials and Methods

### 2.1. Ethics

The ethics committee of Hallym University (23 October 2019) approved this study. The requirement for written informed consent was waived by the Institutional Review Board. All analyses adhered to the guidelines and regulations of the ethics committee of Hallym University.

### 2.2. Study Population and Participant Selection

A detailed description of the Korean National Health Insurance Service-Health Screening Cohort data is described elsewhere [16]. This study retrospectively analyzed this cohort data.

**Figure 1:**
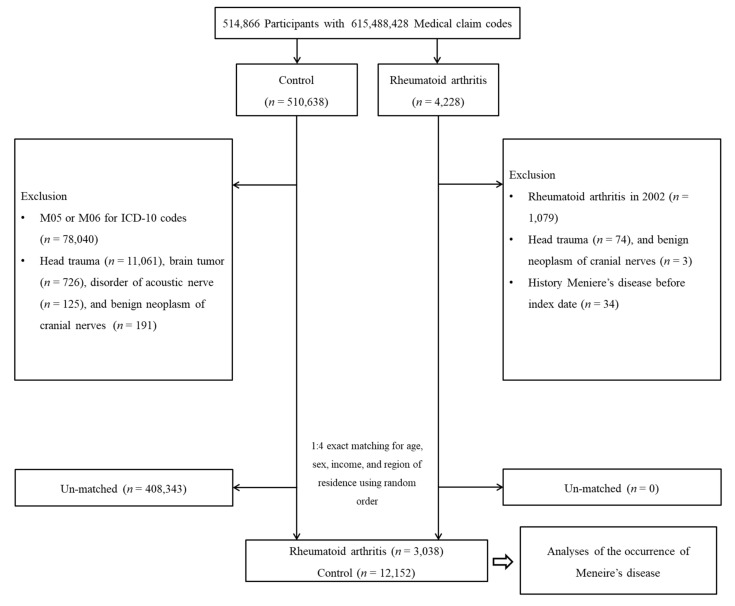
A schematic illustration of the participant selection process that was used in the present study. Among a total of 514,866 participants, 3038 rheumatoid arthritis participants were matched with 12,152 control participants for age, sex, income, and the region of residence.

RA participants were selected from among 514,866 participants with 615,488,428 medical claim codes from 2002 through 2015 (*n* = 4228). The control group was included if participants were not defined as having RA from 2002 through 2015 (*n* = 510,638). To ensure the selection of participants in whom RA was diagnosed first, RA participants diagnosed in 2002 were excluded (washout period, *n* = 1079). Control participants were excluded if they had M05 or M06 International Classification of Diseases, 10th Revision (ICD-10), diagnostic codes (*n* = 78,040). Participants who were treated for head traumas (ICD-10 codes: S00 to S09, diagnosed by neurologists, neurosurgeons, or emergency medicine doctors) ≥2 times and evaluated by head and neck computed tomography (CT; claim codes: HA401-HA416, HA441-HA443, HA451-HA453, HA461-HA463, or HA471-HA473) were excluded (*n* = 74 for the RA group, *n* = 11,061 for the control group). Control participants who were treated for brain tumors (ICD-10 codes: C70 to C72) ≥2 times (*n* = 726) or disorders of the auditory nerve (ICD-10 codes: H933) ≥2 times (*n* = 125) were excluded. Participants who were treated for benign neoplasms of the cranial nerves (ICD-10 codes: D333) ≥2 times (*n* = 3 for the RA group, *n* = 191 for the control group) were excluded. RA participants were 1:4 matched with control participants in terms of age, sex, income, and the region of residence. To avoid selection bias for the selection of matched participants, the control participants were selected in a random number order. The index date of control participants was identically set as that of the matched RA participants. In both the RA and control groups, participants who had a history of MD before the index date were excluded. In the RA group, 34 participants were excluded. During the matching procedure, 408,343 control participants were excluded. Finally, 3038 RA participants were 1:4 matched with 12,152 control participants (Figure 1)

### 2.3. Definition of RA

RA was defined as the presence of ICD-10 codes M05 or M06 and a prescription for a biologic agent or any disease-modifying anti-rheumatic drug (DMARD) [17].

### 2.4. Definition of MD

MD was defined as the presence of ICD-10 code H810 (MD). Among participants with this code, we selected those who were treated ≥2 times and underwent an audiometric examination (claim code: E6931-E6937, F6341-F6348) [18].

### 2.5. Covariates

Age groups were divided into 10 groups based on 5-year intervals: 40–44, and 85+ years. Income was classified into 5 classes (class 1 (lowest income)–5 (highest income)). The region of residence was classified as urban or rural areas following our previous study [19]. Tobacco smoking, alcohol consumption, obesity (defined using body mass index (BMI, kg/m^2^) based on the Asia-Pacific criteria [20]), systolic blood pressure (SBP, mmHg), diastolic blood pressure (DBP, mmHg), fasting blood glucose (mg/dL), and total cholesterol (mg/dL) were surveyed or measured. The Charlson Comorbidity Index (CCI) score without rheumatic diseases was calculated. The comorbidities were retrieved based on diagnostic codes (ICD-10).

### 2.6. Statistical Analyses

The standardized difference (SD) was used to compare the rate of general characteristics between the RA and control groups [21]. The SD is an index to measure the effect size between two groups. Because SD is independent of sample size, it can compare baseline covariates in large cohort data.

Stratified Cox proportional hazard models were used to assess the hazard ratios (HRs) and 95% confidence intervals (CIs) of RA for participants with MD. Crude (simple) and adjusted (obesity, smoking, alcohol consumption, SBP, DBP, fasting blood glucose, total cholesterol, CCI scores, benign paroxysmal vertigo, vestibular neuronitis, and other peripheral vertigo) models were used in these analyses, and the 95% CI was calculated. In these analyses, age, sex, income, and the region of residence were stratified. A Kaplan–Meier curve and log rank test were used.

For the subgroup analyses using the stratified Cox proportional hazards model, we divided participants by age (<60 years old and ≥60 years old), sex, income, and the region of residence.

We performed additional subgroup analyses using the unstratified Cox proportional hazards model and analyzed additional subgroup analyses.

Two-tailed analyses were performed, and significance was defined as *p* values of less than 0.05. SAS version 9.4 (SAS Institute Inc., Cary, NC, USA) was used for statistical analyses.

## 3. Results

A total of 1.5% (45/3038) of RA and 1.3% (163/12,152) of control groups had histories of MD (SD = 0.01, Table 1). The RA group and control group had no significant difference in the rates of histories of benign paroxysmal vertigo (8.5% vs. 6.6%, SD = 0.07), vestibular neuronitis (2.1% vs. 1.6%, SD = 0.04), or other peripheral vertigo (5.1% vs. 4.5%, SD = 0.03). The distributions of obesity, smoking status, alcohol consumption, SBP, DBP, fasting blood glucose, total cholesterol, and CCI scores were not significantly different between the RA and control groups.

A history of RA was not associated with MD in either the crude model or the adjusted model (adjusted HR (aHR) = 1.03, 95% CI = 0.73–1.44, *p* = 0.885, Table 2). A higher HR of RA for participants with MD was found in the ≥ 60-year-old subgroup in the crude model (HR = 1.57, 95% CI = 1.03–2.40, *p* = 0.035) but not in the adjusted model (aHR = 1.40, 95% CI = 0.91–2.15, *p* = 0.131).

The analysis of the subgroups of participants < 60 years old, men, women, participants with low income, participants with high income, urban residents, and rural residents did not show a significant association of RA with MD in either the crude model or the adjusted model (all *p* > 0.05 and P for interaction > 0.05, Table 3). The analysis of additional subgroups of patients with the comorbidities of obesity, smoking, alcohol consumption, blood pressure, fasting blood glucose, total cholesterol, CCI score, benign paroxysmal vertigo, vestibular neuronitis, and other peripheral vertigo did not demonstrate a significant association of RA with MD in either the crude model or the adjusted model (all *p* > 0.05 and P for interaction > 0.05).

## 4. Discussion

We could not determine whether pre-existing RA may increase the risk of MD in the adult population. Although the older population (>60 years old) showed a higher risk of MD associated with RA in the crude analysis, the association of prior RA with MD was not evident after adjusting for other possible confounders. This study improved previous findings on the potential association of RA with MD by using a large population cohort and considering many confounders, including past medical histories, lifestyle factors, and other vestibular disorders. Moreover, comprehensive subgroup analyses were conducted and identified no significant differential contribution of demographic factors, comorbidities, or other vestibular disorders to the relation of RA with MD.

The association of RA with MD has been controversial. Several previous studies suggested the association of RA with MD [13,22,23]. In a systematic review, the average point prevalence of RA was estimated to be approximately 4.7% (range = 1.0–10.0%) in patients with MD [13], which was higher than the prevalence of MD in the general population (0–1.1%) [13]. In addition, the symptomatic progression of hearing loss and vertigo in MD were correlated with increased levels of lymphocytes in systemic autoimmune diseases (R^2^ = 0.52, *p* = 0.01 for hearing loss with CD19 cells and R^2^ = 0.12, *p* = 0.05 for hearing loss CD8 cells) [22]. Among 41 patients with MD, 20% of patients showed autoimmunity to type II collagen, and these patients demonstrated a higher rate of the specific HLA-DRB1 genotype of HLA-DRB1*0405 than other patients (63% vs. 12%) [23]. This HLA-DRB1*0405 genotype is a susceptible genotype for RA, which implies shared genetic susceptibility to both MD and RA [23]. However, most previous studies lacked a control population and had a small study population. In addition, these previous studies might not have appropriately adjusted for confounders. In a large population study with a control population, the potential association of RA with MD was not strong enough to show statistical significance, as shown in the present results. 

There was no significant association between RA and MD in the present study. Because comorbidities, including other vestibular disorders and lifestyle factors, were adjusted for in the present study, the mediating effects of these factors may have been alleviated in this study. Conversely, the confounders that were not adjusted for may have mediated the links between RA and MD in previous studies. In the older age subgroup, a previous history of RA was positively related to MD before adjusting for other variables in the present study. The high prevalence of comorbidities in the older population could impose additional associations between RA and MD before adjusting for these confounding factors. Moreover, the modulation of autoimmunity by medications for RA, including DMARDs, could have attenuated the impact of RA on the new onset of MD in the present study. A case report demonstrated that a patient with RA suffered from vestibular vertigo symptoms, which was attributed to immunosuppression by anti-rheumatic medication with a DMARD [24]. In addition, patients with RA can be treated with glucocorticoids as an adjunct treatment to conventional DMARDs. Because systemic glucocorticoids are also used in refractory MD, this could be another possible confounder in this study. Medication histories and measures of disease activities were not available in the present study. Future studies with information on the management of RA will be warranted to unravel the impact of RA management on the occurrence of MD.

The association of RA with MD may be limited to certain types of MD, such as autoimmune-mediated MD. Although autoimmunity is one of the pathophysiologic factors associated with the occurrence of MD [10], other pathophysiologic factors have been proposed to underlie the occurrence of MD, such as inflammation, viral infection [25], allergy [26], and genetic factors [11,27]. For instance, a retrospective study reported a higher prevalence of RA and autoimmune disease in patients with familial MD than in patients with sporadic MD (16.9% vs. 4.5%, *p* = 0.002) [28]. The proportion of individuals with familial MD was estimated to be only approximately 2.6–23.5% of patients with MD [28]. In addition, other vestibular disorders or central diseases causing vertigo could contribute to vertigo in patients with RA, which could be confused with MD. A prospective study found that among 81 RA patients, 21 patients (24.69%) showed central vertigo, five patients (6.17%) showed peripheral vertigo, and six patients (7.4%) showed mixed-type vertigo in videonystagmography tests [29]. A few case reports described vertebral artery insufficiency or occlusion causing recurrent vertigo in patients with RA [30,31]. Because the present cohort included participants with all types of MD, the impact of RA on MD might not be considerable.

This study included 3038 RA patients, which is one of the largest cohort studies on RA. Data on many covariates, including demographic factors, lifestyle factors, chronic diseases, and other vestibular disorders, were collected, and these covariates were adjusted for to minimize possible confounding effects. However, the number of patients with MD was not sufficient (*n* = 208), which could have contributed to the nonsignificant association of RA with MD found in the present study. In addition, the severities and durations of RA and MD were heterogeneous in our cohort. Because this study used health claim data, the laboratory findings of autoimmune antibodies and the results of vestibular function tests were not available. Although this study excluded the patients with other vestibular disorders, otologic diseases which could be an etiology of MD, such as acoustic trauma and otitis media, were not excluded. However, both RA and MD were diagnosed by clinical physicians, and participants had treatment histories for their diseases; individuals with subclinical or mild cases were excluded in this study. Although numerous variables were adjusted for, other confounders that were not analyzed in this study, such as stress, nutritional status, and physical activity, may have had an effect on the results.

## 5. Conclusions

A prior history of RA was not associated with an increased risk of MD in the adult population. Although the higher risk of MD related with RA was demonstrated in old age population in univariate analysis, the association of RA with MD was not valid after considering other comorbidities, including other vestibular disorders.

## Figures and Tables

**Table 1:** General Characteristics of Participants.

Characteristics		Total Participants		
		Rheumatoid Arthritis (*n*, %)	Control (*n*, %)	Standardized Difference
Age (years old)				0.00
	40–44	106 (3.5)	424 (3.5)	
	45–49	355 (11.7)	1420 (11.7)	
	50–54	658 (21.7)	2632 (21.7)	
	55–59	574 (18.9)	2296 (18.9)	
	60–64	532 (17.5)	2128 (17.5)	
	65–69	417 (13.7)	1668 (13.7)	
	70–74	238 (7.8)	952 (7.8)	
	75–79	120 (4.0)	480 (4.0)	
	80–84	33 (1.1)	132 (1.1)	
	85+	5 (0.2)	20 (0.2)	
Sex				0.00
	Male	815 (26.8)	3260 (26.8)	
	Female	2223 (73.2)	8892 (73.2)	
Income				0.00
	1 (lowest)	509 (16.8)	2036 (16.8)	
	2	465 (15.3)	1860 (15.3)	
	3	514 (16.9)	2056 (16.9)	
	4	654 (21.5)	2616 (21.5)	
	5 (highest)	896 (29.5)	3584 (29.5)	
Region of residence				0.00
	Urban	1311 (43.2)	5244 (43.2)	
	Rural	1727 (56.9)	6908 (56.9)	
Obesity †				0.05
	Underweight	56 (1.8)	276 (2.3)	
	Normal	1156 (38.1)	4437 (36.5)	
	Overweight	794 (26.1)	3269 (26.9)	
	Obese I	942 (31.0)	3774 (31.1)	
	Obese II	90 (3.0)	396 (3.3)	
Smoking status				0.04
	Nonsmoker	2497 (82.2)	10,167 (83.7)	
	Past smoker	208 (6.9)	802 (6.6)	
	Current smoker	333 (11.0)	1183 (9.7)	
Alcohol consumption				0.04
	<1 time a week	2452 (80.7)	9616 (79.1)	
	≥1 time a week	586 (19.3)	2536 (20.9)	
Systolic blood pressure				0.07
	<120 mmHg	986 (32.5)	3863 (31.8)	
	120–139 mmHg	1474 (48.5)	5632 (46.4)	
	≥140 mmHg	578 (19.0)	2657 (21.9)	
Diastolic blood pressure				0.06
	<80 mmHg	1497 (49.3)	5648 (46.5)	
	80–89 mmHg	1037 (34.1)	4265 (35.1)	
	≥90 mmHg	504 (16.6)	2239 (18.4)	
Fasting blood glucose				0.08
	<100 mg/dL	2125 (70.0)	8061 (66.3)	
	100–125 mg/dL	710 (23.4)	3134 (25.8)	
	≥126 mg/dL	203 (6.7)	957 (7.9)	
Total cholesterol				0.04
	<200 mg/dL	1583 (52.1)	6128 (50.4)	
	200–239 mg/dL	1019 (33.5)	4147 (34.1)	
	≥240 mg/dL	436 (14.4)	1877 (15.5)	
CCI score				0.18
	0	1989 (65.5)	8827 (72.6)	
	1	556 (18.3)	1508 (12.4)	
	≥2	493 (16.2)	1817 (15.0)	
Benign paroxysmal vertigo		258 (8.5)	796 (6.6)	0.07
Vestibular neuronitis		65 (2.1)	190 (1.6)	0.04
Other peripheral vertigo		156 (5.1)	550 (4.5)	0.03
Meniere’s disease		45 (1.5)	163 (1.3)	0.01

Abbreviations: CCI, Charlson comorbidity index. † Obesity (BMI, body mass index, kg/m^2^) was categorized as <18.5 (underweight), ≥18.5 to <23 (normal), ≥23 to <25 (overweight), ≥25 to <30 (obese I), and ≥30 (obese II).

**Table 2:** Crude and adjusted hazard ratios of RA for Meniere’s disease with subgroup according to age, sex, income, and region.

Independent Variables		Meniere’s Disease/Participants (*n*, %)	Follow-Up Duration (PY)	IR Per 10,000(PY)	Hazard Ratios for Meniere’s Disease (95% Confidence Interval)				P for Interaction
					Crude †	*p* Value	Adjusted †‡	*p* Value	
Total participants (*n* = 15,190)									
	RA	45/3038 (1.5)	21,095	21.3	1.13 (0.81–1.57)	0.473	1.03 (0.73–1.44)	0.885	
	Control	163/12,152 (1.3)	84,712	19.2	1		1		
Age < 60 (*n* = 8465)									0.054
	RA	15/1693 (0.9)	13,145	11.4	0.72 (0.42–1.25)	0.243	0.68 (0.39–1.18)	0.168	
	Control	84/6772 (1.2)	52,490	16.0	1		1		
Age ≥ 60 (*n* = 6725)									
	RA	30/1345 (2.2)	7950	37.7	1.57 (1.03–2.40)	0.035 *	1.40 (0.91–2.15)	0.131	
	Control	79/5380 (1.5)	32,222	24.5	1		1		
Men (*n* = 4075)									0.605
	RA	5/815 (0.6)	4991	10.0	0.87 (0.33–2.29)	0.782	0.82 (0.29–2.29)	0.703	
	Control	25/3260 (0.8)	20,348	12.3	1		1		
Women (*n* =11,115)									
	RA	40/2223 (1.8)	16,104	24.8	1.17 (0.82–1.67)	0.377	1.06 (0.74–1.52)	0.737	
	Control	138/8892 (1.6)	64,364	21.4	1		1		
Low income (*n* = 7440)									0.279
	RA	16/1488 (1.1)	10,528	15.2	0.88 (0.51–1.51)	0.633	0.81 (0.47–1.40)	0.443	
	Control	75/5952 (1.3)	42,219	17.8	1		1		
High income (*n* = 7750)									
	RA	29/1550 (1.9)	10,567	27.4	1.34 (0.88–2.04)	0.171	1.20 (0.78–1.84)	0.410	
	Control	88/6200 (1.4)	42,493	20.7	1		1		
Urban residents (*n* = 6555)									0.723
	RA	20/1311 (1.5)	9257	21.6	1.12 (0.68–1.84)	0.647	0.99 (0.59–1.64)	0.957	
	Control	72/5244 (1.4)	37,291	19.3	1		1		
Rural residents (*n* = 8635)									
	RA	25/1727 (1.4)	11,838	21.1	1.13 (0.73–1.77)	0.580	1.09 (0.69–1.71)	0.715	
	Control	91/6908 (1.3)	47,421	19.2	1		1		

Abbreviations: RA, rheumatoid arthritis; IR, incidence rate. † Models were stratified by age, sex, income, and region of residence. ‡ The model was adjusted for obesity, smoking, alcohol consumption, systolic blood pressure, diastolic blood pressure, fasting blood glucose, total cholesterol, CCI scores, benign paroxysmal vertigo, vestibular neuronitis, and other peripheral vertigo. * Stratified cox proportional hazard regression model; significance at *p* < 0.05.

**Table 3:** Subgroup analyses of crude and adjusted hazard ratios of RA for Meniere’s disease according to obesity, smoking, alcohol consumption, blood pressure, fasting blood glucose, total cholesterol, CCI scores, benign paroxysmal vertigo, vestibular neuronitis, and other peripheral vertigo.

	Independent Variables	Meniere’s Disease/Participants (*n*, %)	Follow-Up Duration (PY)	IR Per (PY)	Hazard Ratios for Meniere’s Disease (95% Confidence Interval)				P for Interaction
					Crude	*p* Value	Adjusted †	*p* Value	
Obesity									0.942
	Underweight (*n* = 332)								
		RA	0/56 (0.0)	363	0.0	N/A		N/A	
		Control	4/276 (1.4)	1815	22.0	1		1	
	Normal weight (*n* = 5593)								
		RA	14/1156 (1.2)	8006	17.5	1.09 (0.60–1.97)	0.775	1.02 (0.55–1.86)	0.962
		Control	50/4437 (1.1)	31,170	16.0	1		1	
	Overweight (*n* = 4063)								
		RA	14/794 (1.8)	5561	25.2	1.21 (0.67–2.20)	0.525	1.09 (0.59–1.99)	0.787
		Control	47/3269 (1.4)	22,587	20.8	1		1	
	Obese (*n* = 5202)								
		RA	17/1032 (1.6)	7165	23.7	1.11 (0.65–1.91)	0.692	0.98 (0.57–1.68)	0.934
		Control	62/4170 (1.5)	29,140	21.3	1		1	
Smoking									0.338
	Non-smoker (*n* = 12,664)								
		RA	42/2497 (1.7)	17,915	23.4	1.16 (0.83–1.64)	0.386	1.04 (0.73–1.46)	0.842
		Control	146/10,167 (1.4)	72,454	20.2	1		1	
	Past smoker and current smoker (*n* = 2526)								
		RA	3/541 (0.6)	3180	9.4	0.67 (0.20–2.30)	0.529	0.68 (0.19–2.41)	0.546
		Control	17/1985 (0.9)	12,258	13.9	1		1	
Alcohol consumption									0.462
	<1 time a week (*n* = 12,068)								
		RA	42/2452 (1.7)	17,995	23.3	1.14 (0.81–1.61)	0.450	1.02 (0.72–1.45)	0.898
		Control	145/9616 (1.5)	70,869	20.5	1		1	
	≥1 time a week (*n* = 3122)								
		RA	3/586 (0.5)	3100	9.7	0.74 (0.22–2.50)	0.625	0.59 (0.16–2.12)	0.415
		Control	18/2536 (0.7)	13,843	13.0	1		1	
Blood pressure									0.345
	Systolic blood pressure < 140 mmHg and diastolic blood pressure < 90 mmHg (*n* = 11,248)								
		RA	37/2323 (1.6)	15,659	23.6	1.20 (0.83–1.74)	0.328	1.08 (0.75–1.57)	0.682
		Control	119/8925 (1.3)	60,520	19.7	1		1	
	Systolic blood pressure ≥ 140 mmHg or diastolic blood pressure ≥ 90 mmHg (*n* = 3942)								
		RA	8/715 (1.1)	5436	14.7	0.81 (0.38–1.73)	0.592	0.73 (0.34–1.55)	0.409
		Control	44/3227 (1.4)	24,192	18.2	1		1	
Fasting blood glucose									0.147
	<100 mg/dL (*n* = 10,186)								
		RA	31/2125 (1.5)	15,284	20.3	1.00 (0.67–1.48)	0.982	0.87 (0.58–1.30)	0.499
		Control	118/8061 (1.5)	57,842	20.4	1		1	
	≥100 mg/dL (*n* = 5004)								
		RA	14/913 (1.5)	5811	24.1	1.44 (0.79–2.62)	0.235	1.36 (0.74–2.49)	0.329
		Control	45/4091 (1.1)	26,870	16.7	1		1	
Total cholesterol									0.898
	<200 mg/dL (*n* = 7711)								
		RA	22/1583 (1.4)	10,808	20.4	1.13 (0.70–1.81)	0.624	1.02 (0.63–1.65)	0.940
		Control	76/6128 (1.2)	42,013	18.1	1		1	
	≥200 mg/dL (*n* = 7479)								
		RA	23/1455 (1.6)	10,287	22.4	1.10 (0.69–1.74)	0.693	0.99 (0.62–1.57)	0.957
		Control	87/6024 (1.4)	42,699	20.4	1		1	
CCI score									0.619
	0 (*n* = 10,816)								
		RA	24/1989 (1.2)	13,915	17.2	0.97 (0.63–1.51)	0.899	0.89 (0.57–1.39)	0.603
		Control	110/8827 (1.2)	61,949	17.8	1		1	
	1 (*n* = 2064)								
		RA	11/556 (2.0)	3825	28.8	1.14 (0.57–2.30)	0.716	1.14 (0.55–2.36)	0.717
		Control	27/1508 (1.8)	10,825	24.9	1		1	
	≥2 (*n* = 2310)								
		RA	10/493 (2.0)	3355	29.8	1.37 (0.66–2.85)	0.395	1.13 (0.53–2.40)	0.761
		Control	26/1817 (1.4)	11,938	21.8	1		1	
Benign paroxysmal vertigo									0.125
	No (*n* = 14,136)								
		RA	24/2780 (0.9)	19,294	12.4	0.84 (0.54–1.30)	0.422	0.82 (0.53–1.27)	0.363
		Control	118/11,356 (1.0)	79,202	14.9	1		1	
	Yes (*n* = 1054)								
		RA	21/258 (8.1)	1801	116.6	1.44 (0.86–2.42)	0.166	1.48 (0.87–2.51)	0.146
		Control	45/796 (5.7)	5510	81.7	1		1	
Vestibular neuronitis									0.372
	No (*n* = 14,935)								
		RA	38/2973 (1.3)	20,637	18.4	1.02 (0.72–1.46)	0.897	0.93 (0.65–1.34)	0.707
		Control	150/11,962 (1.3)	83,376	18.0	1		1	
	Yes (*n* = 255)								
		RA	7/65 (10.8)	458	152.8	1.64 (0.65–4.12)	0.294	1.78 (0.65–4.82)	0.260
		Control	13/190 (6.8)	1336	97.3	1		1	
Other peripheral vertigo									0.322
	No (*n* = 14,484)								
		RA	33/2882 (1.1)	19,956	16.5	1.00 (0.68–1.46)	0.985	0.90 (0.61–1.32)	0.591
		Control	134/11,602 (1.2)	80,665	16.6	1		1	
	Yes (*n* = 706)								
		RA	12/156 (7.7)	1139	105.4	1.46 (0.75–2.87)	0.268	1.29 (0.64–2.62)	0.477
		Control	29/550 (5.3)	4047	71.7	1		1	

Abbreviations: RA, rheumatoid arthritis; IR, incidence rate. † The model was adjusted for age, sex, income, region of residence, obesity, smoking, alcohol consumption, systolic blood pressure, diastolic blood pressure, fasting blood glucose, total cholesterol, CCI scores, benign paroxysmal vertigo, vestibular neuronitis, and other peripheral vertigo.

## Data Availability

Releasing of the data by the researcher is not legally permitted. All data are available from the database of the Korea Center for Disease Control and Prevention. The Korea Center for Disease Control and Prevention allows data access, at a particular cost, for any researcher who promises to follow the research ethics. The data of this article can be downloaded from the website after agreeing to follow the research ethics.
